# Microbiota-Driven Strategies for Managing IBD-Associated Risks: From Infections to Mental Health

**DOI:** 10.3390/ph19010118

**Published:** 2026-01-09

**Authors:** Patrycja Krynicka, Pablo Cortegoso Valdivia, Maciej Morawski, Wojciech Marlicz, Karolina Skonieczna-Żydecka, Anastasios Koulaouzidis

**Affiliations:** 1 Department of Gastroenterology and Nutrition Disorders, Collegium Medicum in Bydgoszcz, Nicolaus Copernicus University, 87-100 Bydgoszcz, Poland; krynickapatrycja@gmail.com; 2 Gastroenterology and Endoscopy Unit, University Hospital of Parma, 43126 Parma, Italy; cortegosopablo@yahoo.it; 3 Department of Clinical Research, University of Southern Denmark, 5230 Odense, Denmark; 4 Surgical Research Unit, Odense University Hospital og Svendborg Sygehus, 5700 Svendborg, Denmark; 5 Clinical Department of Ophthalmology, 10th Military Clinical Hospital with Polyclinic, 85-681 Bydgoszcz, Poland; maciej.morawski9@gmail.com; 6 Department of Gastroenterology, Pomeranian Medical University, 70-204 Szczecin, Poland; wojciech.marlicz@sanprobi.pl; 7 Department of Human Nutrition and Metabolomics, Pomeranian Medical University, 71-460 Szczecin, Poland; karolina.skonieczna.zydecka@pum.edu.pl

**Keywords:** inflammatory bowel disease (IBD), gut microbiota, fecal microbiota transplantation (FMT), live biotherapeutic products (LBPs), psychobiotics, extraintestinal manifestations (EIMs)

## Abstract

Inflammatory bowel diseases (IBD) are increasingly acknowledged not merely as confined gastrointestinal disorders but as systemic immunometabolic syndromes. Central to this paradigm is the gut microbiota including non-bacterial components such as the virome, whose functional disruption marked by reduced short-chain fatty acids (SCFAs), increasingly implicated in pathogenic processes extending beyond intestinal mucosa. This review outlines how these alternations compromise the epithelial barrier and immune regulation, increasing the risk of recurrent *Clostridioides difficile* infections to anemia, neuropsychiatric comorbidities, and extraintestinal manifestations. We critically evaluate emerging microbiota-targeted strategies, including fecal microbiota transplantation (FMT), live biotherapeutic products (LBPs), and precision postbiotics, positioning them as potential adjuncts to conventional immunosuppression. Finally, we discuss the current barriers to clinical translation, such as safety and heterogeneity, and propose a future framework for personalized, functionally integrated IBD care aimed at restoring long-term microbiota homeostasis.

## 1. Introduction

Inflammatory bowel diseases (IBD), including Crohn’s disease (CD) and ulcerative colitis (UC), are chronic, relapsing-remitting gastrointestinal tract disorders with emerging global incidence and expanding healthcare costs. During the last two decades, the epidemiology of IBD changed profoundly, not only in the form of the rising incidence and prevalence of the disease all over the world but also as a result of increased awareness of the systemic comorbidities, late-onset cases, and intricate extraintestinal manifestations (EIMs) [1]. Notably, in a subset of patients, systemic comorbidities may precede overt gastrointestinal symptoms, and the dominant phenotype may reflect individual physiological and organ-specific vulnerabilities [2]. Traditionally considered mucosal inflammation disorders, IBD is now increasingly considered a multisystem condition in which dynamic interactions among host immunity, environmental exposures, and the gut microbiota play an important role.

In the new vision, the intestinal microbiota does not present as a mere passive observer but rather as a modifiable controller of host homeostasis, being involved in immune tolerance, metabolic signaling, epithelial integrity, and even neurobehavioral regulation. Disruption of the gut microbial ecosystem broadly termed dysbiosis is a hallmark of IBD. Importantly, dysbiosis also involves non-bacterial components of the gut ecosystem, including the virome and mycobiome, which can shape microbial community dynamics and modulate the mucosal immune response [3]. However, the concept of “dysbiosis” has limited clinical utility unless refined into specific, actionable disruptions in microbial function. Recent research emphasizes that microbial metabolites, including short-chain fatty acids (SCFAs), bile acids (BAs) derivatives, and tryptophan catabolites, play central roles in the control of inflammation, barrier integrity, and systemic immune responses [4]. Importantly, these metabolic pathways mediated by the microbiota influence beyond the gut, modulating host physiology at multiple organ systems.

As examples, secondary BAs suppress opportunistic pathogens like *Clostridioides difficile*, indole derivatives determine mucosal immune tolerance through aryl hydrocarbon receptor (AHR)/pregnane X receptor (PXR) and SCFAs strengthen epithelial barrier function while influencing Treg development and neuroimmune interactions [5]. This growing appreciation has accelerated innovation in next-generation microbiome-based therapeutics, including live biotherapeutic products (LBPs) such as SER-109 and REBYOTA^®^, dietary and postbiotic therapeutics, and precision modulation of microbial metabolites [6]. These strategies offer novel options to reduce disease-associated risks and support host resilience in IBD not only by targeting mucosal inflammation, but also by addressing complications such as recurrent infections, iron deficiency, mental health disturbances, and extraintestinal immune dysfunction [7]. The scientific literature on IBD and the microbiota is expanding rapidly, but existing reviews focus on single mechanisms and fail to provide practical clinical implications. A coherent approach that links the microbiota-dependent metabolome with systemic complications in IBD and identifies which components can be realistically influenced is lacking. The objective of this review is to reframe the discussion on gut microbiota in IBD around the concept of modulable risk axes, defined as clinically relevant, microbiota-linked pathways contributing to IBD-associated morbidity.

By synthesizing recent findings from microbiome research, immunometabolism, and clinical gastroenterology, we aim to offer a functionally integrated, clinically applicable perspective for risk mitigation in IBD through microbiota-targeted strategies.

## 2. Gut Microbiota in IBD: From Dysbiosis to Functional Disruption

### 2.1. Functional Dysbiosis in IBD: From Taxonomy to Metabolic Signatures

In IBD, dysbiosis is often described as a change in the composition of gut bacteria. This leads to a decrease in the number of beneficial bacteria such as *Faecalibacterium prausnitzii* and *Roseburia* spp. and a simultaneous increase in potentially harmful microorganisms such as *Escherichia coli* and *Ruminococcus gnavus* [8]. It is worth noting that dysbiosis can also reflect the selective strains useful for corrective intervention while reducing others, but this usually coincides with reduced biodiversity and ecological resilience, ultimately weakening the regulatory capacity of the microbiome. These changes are often observed, but increasing attention is now being paid to microbiota dysfunction, which may better reflect how microorganisms influence disease variability [9]. Key functional consequences include reduced SCFAs production (discussed in detail in Section 2.4) and altered immunometabolic signaling [10,11]. Abnormal metabolism of tryptophan, an amino acid that under normal conditions is converted by the microbiota into indole derivatives, is also observed. These substances are responsible for supporting the production of IL-22, which is responsible for maintaining a tight mucosal barrier [12]. When these mechanisms are disrupted, susceptibility to inflammation increases and immune tolerance disorders occur [13]. Dysbiosis can also result in increased migration of signaling molecules, such as lipopolysaccharide (LPS) or bacterial flagellin, to the mucosal membrane. This phenomenon leads to the activation of innate immune receptors, which results in the activation of inflammatory pathways such as NF-κB and, ultimately, the production of pro-inflammatory cytokines such as IL-6, IL-1β, and TNF-α [14]. These mechanisms contribute to the development of chronic inflammation and the activation of the entire immune system. As a conclusion, in IBD, it is not only the structure of the microbiota that is important, but also its activity. The human microbiome must be considered as a varying metabolomic system that works in cooperation with the body. Understanding this approach is the foundation for new therapeutic strategies, such as prebiotics, postbiotics, and targeted probiotic therapy, aimed at restoring the proper function of the microbiota. Focusing on dysbiosis as a functional target may offer a more precise framework than approaches aimed solely at altering microbiota composition. This opens up possibilities for more individualized IBD therapy.

### 2.2. The Bile Acid–Microbiota–Immunity Axis

The BAs are increasingly perceived not only as essential substances for fat digestion, but also as signaling molecules with many functions which affect various processes in the body. They are produced in the liver as primary BAs, which are then converted by intestinal bacteria into their secondary forms. These microbiome metabolites play a significant role in regulating the immune system, the integrity of the intestinal barrier, inflammatory processes, and host metabolism [15]. In people with inflammatory bowel disease, this process is often disrupted because dysbiosis leads to a decrease in the number of certain bacteria—especially *Clostridium scindens* which are responsible for converting primary BAs into their secondary forms. In this process, the enzyme 7α-dehydroxylase is involved. A deficiency of this enzyme leads to lower levels of secondary BAs, such as deoxycholic acid (DCA) and lithocholic acid (LCA), which has serious consequences for the balance in the intestines [16]. One of the results of this disorder is a weakening of the activation of BAs receptors, particularly FXR and TGR5. FXR is responsible for the production of antimicrobial peptides and proteins that support the formation of tight junctions in the intestinal epithelium, while TGR5 controls epithelial renewal and cytokine production by immune system cells such as macrophages and dendritic cells. When the stimulation of these receptors is limited, the intestinal barrier is weakened, the regeneration of the mucous membrane is disrupted, and a pro-inflammatory environment is created in the intestines [17]. Additionally, BAs-related signaling pathways affect T cell balance, promoting the development of regulatory Treg and inhibiting inflammation associated with Th17 cells. Preclinical and mechanistic studies suggest that some BAs metabolites, such as isoDCA or 3-oxoLCA, can directly affect the function of immune system cells through specific receptors [15]. In this way, disturbances in BAs metabolism in the context of IBD contribute to both epithelial damage and loss of immune tolerance and chronic inflammation. Preliminary research supports these mechanisms. In models of intestinal inflammation in mice, it was found that restoring the appropriate level of secondary BAs by administering the appropriate bacteria or their metabolites leads to a reduction in inflammation, improvement in barrier function, and restoration of immune system balance [16]. This opens up new possibilities for using postbiotics, or specific bacterial metabolites, as innovative therapeutic strategies for IBD. In summary, the combination of BAs, microbiota, and the immune system is a key area of interaction between the host and its microbiome. Disruptions in this system not only help to understand why the disease can persist despite treatment, but also point to new, promising therapeutic directions aimed at restoring balance in the gut.

### 2.3. The Tryptophan–Indole–AHR/PXR Axis

Tryptophan is one of the key aromatic amino acids that must be provided through diet, as the body cannot produce it on its own. Although it is mainly known as a building block of proteins, tryptophan also plays a very important role as a substrate for the intestinal microbiota. Intestinal bacteria convert it into a series of derivative compounds, such as indole-3-propionic acid (IPA), indole-lactic acid (ILA), or indole-3-aldehyde (IAld), which influence immunity and maintain the integrity of the epithelium [18]. Microbial conversion of tryptophan into indole derivatives may act as a compensatory, tissue-protective mechanism that supports epithelial repair. This process can be inadequate or dysregulated during active IBD. Tryptophan is a shared, finite substrate, greater microbial diversion toward indole production may reduce its availability for host pathways such as serotonin, melatonin, and kynurenine metabolism. However, this repair program is likely context-dependent and may be less effective under low microbial diversity and chronic low-grade inflammation. Indole metabolites interact with the body primarily through the AHR and PXR, which play crucial roles in gene expression and detoxification processes. Activation of AHR supports the expression of genes responsible for the production of antimicrobial peptides, epithelial regeneration, and IL-22 signaling, a key cytokine in mucosal defense. More broadly, cytokines are host effector mediators that translate microbiota-derived metabolic cues into systemic immune and neuroimmune regulation. In turn, PXR activates detoxification enzymes, such as CYP3A4 and MDR1, which protect the body from toxins and inflammation [19]. In patients with IBD, especially during the active phase of the disease, indole derivatives are significantly lower in blood and stool [20]. A deficiency of these compounds is associated with a disrupted intestinal barrier, increased microbial penetration through the epithelium, and a weakened immunoregulatory response by limiting lymphocyte Treg differentiation. Animal models have shown that the absence of AHR ligands is associated with increased susceptibility to inflammation and slower mucosal healing [21]. Therapeutic interventions targeting this metabolic pathway have shown promising results in preclinical studies. Adding specific indole metabolites or AHR can reduce pro-inflammatory signaling and support mucosal homeostasis. A well-chosen diet that increases the availability of tryptophan or supports bacteria that produce indole metabolites can also have a positive effect on the course of the disease, as shown in models of colitis in mice [22]. Importantly, tryptophan metabolism is not limited to the intestines. An increasing amount of data indicates that the tryptophan-indole-AHR/PXR axis also plays a crucial role in inflammatory and metabolic diseases outside the gastrointestinal tract such as mood disorders, liver diseases, or neuroinflammation [19]. Therefore, therapies aimed at modulating this pathway may hold potential systemic relevance, particularly in IBD patients with coexisting neuropsychiatric or hepatobiliary comorbidities. In chronic inflammation, an excessive diversion of tryptophan to microbial indole pathways can lead to an increase in potentially harmful indole-derived uremic toxins, contributing to systemic dysfunction beyond the gut. In summary, the microbial conversion of tryptophan into indole compounds is one of the key mechanisms regulating balance in the intestines. When this mechanism is disrupted, as it is in IBD, it may lead to damage to the epithelium, chronic inflammation, and immune tolerance disorders. That is why this course is now an attractive target for new therapeutic strategies that aim not only to treat the gut, but also to improve overall health.

### 2.4. Short-Chain Fatty Acids (SCFAs) and the Epithelial Barrier

SCFAs are some of the most important metabolites produced by gut bacteria as a result of dietary fiber fermentation. Their main producers are anaerobic bacteria, such as *Faecalibacterium prausnitzii, Roseburia* spp., or *Eubacterium* spp. Among SCFAs, we distinguish three main compounds: acetate, propionate, and butyrate. They play a key role in preserving the integrity of the intestinal barrier, regulating immunity, and creating an acidic environment in the intestinal lumen, which makes it difficult for pathogens to develop [23]. Researchers are particularly interested in butyrate, which is the primary energy source for the cells of the large intestine epithelium, thereby supporting the regeneration and maintenance of the integrity of the mucous membrane. This compound increases the expression of proteins responsible for connections between cells, stimulates the secretion of mucin by goblet cells, and strengthens the mucus layer that separates host cells from intestinal bacteria. In patients with IBD, the level of butyrate is usually lower, which is associated with increased intestinal permeability, damaged epithelial barrier, and increased inflammation [24]. However, butyric acid is not only a building material; it also acts as a signaling molecule. It interacts with G protein-coupled receptors (GPCRs), such as GPR41, GPR43, and GPR109A, which are found on epithelial and immune system cells. Through these receptors, butyrate regulates cytokine production and promotes the development of Treg, which are responsible for suppressing undesirable inflammatory reactions and maintaining immune tolerance [25]. In addition, butyrate acts as an inhibitor of histone deacetylases (HDACs), which influences gene expression toward anti-inflammatory reactions. Despite the large therapeutic potential of SCFAs, direct administration in the form of suppositories or oral preparations has so far yielded mixed results. One of the main problems is the rapid absorption of these compounds and their low bioavailability in the distal sections of the large intestine, where inflammation is often located in patients with IBD. Therefore, more attention is being paid to strategies that strive to increase SCFAs production by the microbiome itself, for example, through a proper diet rich in fermentable fibers such as inulin or resistant starch, prebiotics, as well as procedures such as fecal microbiota transplantation (FMT) [26]. Another promising direction is interventions that selectively support butyrate-producing bacteria, for example, through the use of the Mediterranean diet or synbiotic preparations. More research is also being devoted to a new generation of probiotics, such as *Faecalibacterium prausnitzii* or *Roseburia* spp., which aim to restore barrier and immune functions through the production of SCFAs [27]. In summary, SCFAs are a key link between diet, microbiota, and gut health. Their deficiency in IBD patients directly contributes to mucosal damage and chronic inflammation, while their restoration, especially through actions that support the natural microbiota, may be a safe and well-established therapeutic approach to restoring balance in the intestines.

### 2.5. Integration Framework: Barrier–Metabolite–Immunity Loop

**Figure 1:**
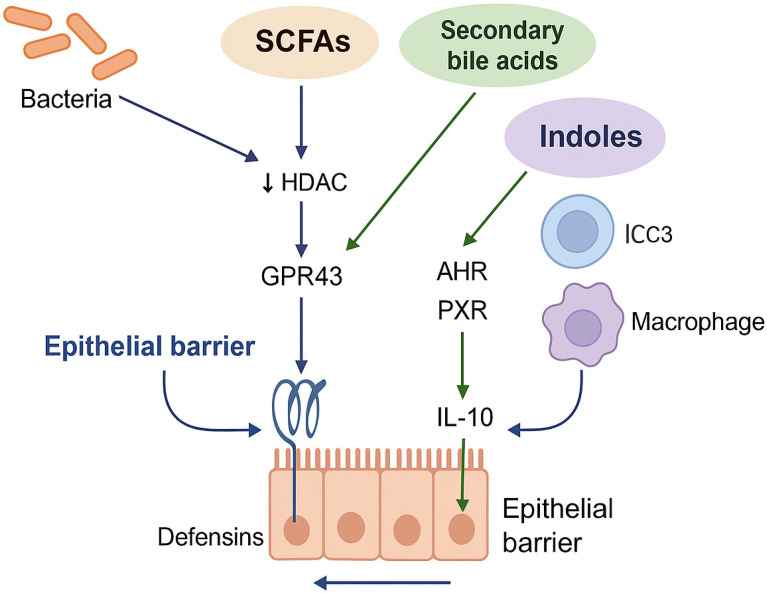
The Barrier–Metabolite–Immunity (BMI) loop in IBD pathogenesis. Short-chain fatty acids (SCFAs), secondary bile acids (BAs), and tryptophan-derived indoles are the three major groups of gut microbial metabolites that influence host physiology. These metabolites act via receptors, including GPR43, GPR109A, HDAC, FXR, TGR5, AHR, and PXR, to regulate epithelial barrier integrity, cytokine production (e.g., IL-10, IL-22), Treg differentiation, and anti-inflammatory responses. Dysbiosis in IBD leads to loss of these functions, promoting epithelial injury, immune dysregulation, and systemic inflammation. Arrows indicate the direction of regulatory and signaling interactions between microbial metabolites, epithelial cells, and immune components; downward arrows denote inhibitory effects (e.g., HDAC inhibition), whereas standard arrows indicate activation or signaling pathways.

Communication between the gut microbiota and the host’s immune system is based on a precisely regulated network of metabolic signals. They determine the integrity of the epithelial barrier, local and systemic immunity, and the intestine’s ability to repel pathogens. In the course of IBD, this network is severely disrupted, not only by changes in the composition of bacteria, but primarily by the loss of key metabolic functions of the microbiota [28]. Among them, three main axes are particularly important: SCFAs, BAs derivatives, and indoles formed from tryptophan. This is how microorganisms influence the physiology of the host. Moreover, these three pathways do not operate independently, but form a synergistic, self-reinforcing system that we conceptualize as the Barrier–Metabolite–Immunity (BMI) loop [29]. In this integrated framework, the failure of one axis inevitably destabilizes the others. For instance, a deficit in SCFAs availability compromises epithelial hypoxia, which is required for the maintenance of anaerobic obligate bacteria that convert bile acids. Consequently, restoring homeostasis requires reactivating the loop as a whole rather than targeting single metabolites in isolation. In the case of dysbiosis, which is observed in patients with IBD, this entire loop is disrupted. Reducing the number of butyrate-producing bacteria, such as *Faecalibacterium prausnitzii* and *Roseburia* spp., leads to a weakening of epithelial cell nutrition and an increase in its permeability [30]. At the same time, a decrease in the level of secondary BAs weakens the FXR/TGR5 signaling and the deficiency of indole metabolites limits the AHR activity, which worsens the barrier’s tightness and suppresses the immunoregulatory response [31]. As a result, there is a translocation of microorganisms or their fragments (pathogen-associated molecular patterns- PAMPs), activation of innate immunity receptors, and increased production of pro-inflammatory cytokines, such as IL-1β, IL-6, and TNF-α. A vicious cycle of mucosal inflammation and systemic immune activation is created, which may contribute to extraintestinal manifestations, such as depression, hepatitis, or metabolic disorders [32]. From a therapeutic standpoint, such a complex mechanism suggests that targeting a single metabolite or pathway may not be sufficient. Effective intervention should aim to restore the full spectrum of microbiota functions and reactivate the entire BMI loop. In this context, promising strategies include multi-strain FMT, engineering consortia, and complex prebiotics and postbiotic formulas that have the ability to influence multiple axes simultaneously [33]. Significant differences in the microbiota and metabolome can be observed in patients with IBD. These differences are due to differences in diet, medications (especially antibiotics and immunosuppression), disease activity, location, and sampling method. Therefore, it is difficult to obtain similar results when studying different groups of IBD patients. Although therapies targeting the microbiota and metabolome are important, they may work better in some patients than in others. Therefore, we need better, standardized assessment methods and biomarkers that can help predict who will benefit most. In summary, IBD can be considered not only as an inflammatory disease but also as a microbiome-metabolic failure syndrome, in which the communication between the microbiota and the host is disrupted. The BMI loop concept helps to understand this complexity and sets new directions for targeted interventions restoring intestinal homeostasis. Table 1 provides a detailed overview of key microbiota-derived metabolites, their microbial sources, molecular targets in the host, and the implications of their disruption in the context of IBD pathogenesis. This integrative concept is illustrated in Figure 1, which highlights how key microbiota-derived metabolites shape the intestinal barrier and immune responses in the BMI loop.

## 3. Clinical Implications of Microbiota Dysfunction in IBD: From Infections to Extraintestinal Manifestations

### 3.1. Infections and Colonization Resistance in IBD

Infectious complications of IBD are a significant burden, and *Clostridioides difficile* infection (CDI) is the most glaring example. IBD patients are at extremely increased risk for primary and recurrent CDI (rCDI), secondary to mucosal inflammation, immune deregulation, and iatrogenic exposures such as antibiotics, immunosuppressives, and proton pump inhibitors (PPI) [34]. One critical underlying mechanism linking IBD with infection susceptibility is the loss of colonization resistance. The ability of the commensal microbiota to suppress the growth of pathogens through nutrient competition, stimulation of immunity, and production of inhibitory metabolites. Functional dysbiosis in IBD is the cause of breakdown of colonization resistance [35]. Decline of butyrate-producing organisms such as *Faecalibacterium prausnitzii* and *Roseburia* spp. leads to a decrease in SCFAs, which are important for epithelial barrier maintenance, mucus production, and local immune regulation [36]. Concurrently, decline of 7α-dehydroxylating bacteria leads to reduced secondary BAs—deoxycholic acid and lithocholic acid—which inhibit *C. difficile* germination and vegetative growth through FXR- and TGR5-mediated mechanisms [37]. IBD also enhances intestinal permeability, which permits translocation of PAMPs such as LPS that continue to drive mucosal inflammation and deplete innate antimicrobial defenses. The clinical consequences of impaired colonization resistance are particularly evident in the context of recurrent CDI. Compared to the overall population, IBD patients have higher levels of rCDI, more severe disease trajectories, greater risk of hospitalization, and worse results after surgery [38]. Of particular importance is that conventional antibiotic therapy often cannot re-establish microbial balance, with recurrence rates of over 30%. This has generated growing interest in microbiota-targeted strategies to re-establish colonization resistance. FMT has emerged as an effective intervention for rCDI, including in IBD patients [39]. Meta-analyses suggest that FMT can achieve clinical cure rates exceeding 80%, although concerns remain regarding its potential to trigger disease flares in up to 10–15% of IBD recipients. In response to the need for greater safety and standardization, LBPs have been developed as next-generation microbiota-based treatments [40]. Among these, SER-109 (marketed as VOWST™) is an oral capsule containing purified *Firmicutes* spores capable of restoring secondary BAs metabolism. In the phase III ECOSPOR III trial, SER-109 achieved an 88% reduction in rCDI recurrence compared to placebo, with a favorable safety profile. Mechanistically, SER-109 enhances colonization resistance by repopulating BAs–modifying organisms, thereby suppressing *C. difficile* outgrowth through restoration of microbial-derived signaling [41]. Another approved product, REBYOTA^®^, is a rectally administered, single-dose suspension containing live microbiota derived from screened donors. Clinical trials have demonstrated its efficacy in preventing rCDI following antibiotic therapy, with durable responses in over 70% of patients and excellent tolerability [42]. The treatment with microbiota-based therapy in IBD is something that should be thoughtfully considered. Decision-making should be driven by disease activity and immunosuppressive therapy and based on recent PPI or antibiotic use. Finally, therapies such as FMT or LBPs also have other therapeutic applications in addition to the prevention of rCDI, including modulation of BAs signaling such as FXR, TGR5, reconstitution of mucosal immunity, and epithelial barrier repair [43]. Future diagnostics, such as measuring secondary BAs levels or SCFAs profiles, may enable clinicians to stratify patients based on their potential for colonization resistance as our understanding of microbial metabolites expands. In this context, microbiota-targeted treatments are a compelling and scientifically supported approach to lowering the risk of infection in IBD. These complex interactions are summarized in Table 2, which outlines the key mechanisms of dysbiosis in IBD, its clinical implications such as recurrent CDI, and the microbiota-based strategies aimed at restoring colonization resistance.

### 3.2. Iron Metabolism and Anemia in IBD: Microbiota Linked Mechanisms and Therapeutic Implications

Anemia is one of the most common systemic complications in patients with IBD. We most often deal with iron deficiency anemia (IDA), which lowers the quality of life, worsens cognitive functions, increases fatigue, and reflects the state of chronic inflammation, malnutrition, and metabolic disorders [44]. Iron deficiency in IBD is caused not only by blood loss from the gastrointestinal tract or impaired absorption in inflamed mucosa, but also, as new studies show, by disruptions in the intestinal microbiota, which plays an important role in regulating iron metabolism. More attention is being paid to the interrelations between iron metabolism, inflammation, and the composition of the microbiome. The key regulator of iron metabolism is hepcidin [45]. This is a peptide synthesized in the liver that blocks the export of iron from cells by degrading the ferroportin protein. Under inflammatory conditions, the overproduction of hepcidin occurs under the influence of cytokines such as IL-6, which leads to the so-called anemia of chronic disease (ACD) [46]. In IBD, additional stimuli that stimulate the production of hepcidin may be microbial components, such as LPS, which activate signaling pathways through Toll-like receptors (TLR). The gut microbiota plays a dual role in iron metabolism. On the one hand, it competes with the host for the availability of iron in the intestinal lumen, and on the other hand, it influences its absorption indirectly by regulating inflammation and the integrity of the mucosa [47]. Dysbiosis typical of IBD, characterized by reduced microbial diversity and impaired metabolic activity, can further exacerbate absorption disorders and maintain inflammation, creating a vicious cycle that leads to the persistence of anemia [48]. The type of iron supplementation used has a significant impact not only on the effectiveness of treatment, but also on the microbiome. Although oral iron preparations are commonly used in milder cases of anemia, they can disrupt the microbiological balance [49]. Research has shown that iron supplementation, especially with iron sulfate, promotes the growth of potentially pathogenic bacteria, such as *Escherichia coli, Salmonella*, or *Clostridioides difficile*, while reducing the number of beneficial bacteria that produce SCFAs. Such a microbiological profile promotes inflammation, oxidative stress, and increased calprotectin in feces [50]. In this situation, intravenous iron preparations such as iron carboxymaltose or iron isomaltoside represent a safer, “microbiota-sparing” strategy [51]. Unlike oral supplements, IV preparations bypass the gastrointestinal lumen entirely, preventing the bloom of pathogens like *E.coli* and preserving the abundance of beneficial butyrate producers. They quickly replenish iron stores and increase hemoglobin levels, regardless of the hepcidin-induced absorption blockade. Therefore, in the presence of active inflammation or known dysbiosis, clinicians might consider a lower threshold for switching to IV iron than traditionally recommended, viewing it not just as a hematologic intervention but as a maneuver to prevent further microbiome deterioration [51]. Clinical and observational studies indicate that IV iron formulations are more effective and better tolerated in the context of active inflammation. Current European Crohn’s and Colitis Organization (ECCO) guidelines recommend their use when Hb levels drop below 10 g/dL or in situations where oral supplementation is ineffective or intolerable [52]. Research is also being conducted on strategies to support the treatment of anemia and limit the negative impact of supplementation on the microbiome. *Lactiplantibacillus plantarum 299v* strain has the ability to improve iron absorption, reduce gastrointestinal symptoms, and maintain microbial balance during supplementation [53]. Moreover, therapies aimed at modulating hepcidin—both pharmacological (e.g., anti-IL-6, anti-TNF drugs) and dietary can improve iron utilization while alleviating inflammation [54]. According to the latest reports, treatment of anemia in IBD should take into account not only hemoglobin and iron stores, but also disease activity, degree of inflammation, microbiota status, and patient tolerance to treatment. Individualized strategies that combine iron supplementation with protection of the intestinal microbiota may contribute not only to improving the hematological parameters but also to better disease control and improved quality of life for patients.

### 3.3. Mental Health and the Gut–Brain–Immune Axis in IBD

The interaction known as the gut–brain–immune axis connects the nervous, endocrine, and immune systems. In the context of IBD, it can affect not only gastrointestinal symptoms but also the development of comorbid psychiatric disorders, such as depression and anxiety. Epidemiological studies clearly show that patients with IBD have a significantly higher risk of mood disorders. Depression affects approximately 25–35% of patients, and anxiety symptoms even 30–40%, which is much more common than in the general population [55]. Importantly, these disorders are not just a reaction to chronic disease. It is increasingly being pointed out that they share common pathophysiological mechanisms with IBD, including inflammation, a damaged intestinal barrier, and disrupted microbiota metabolic signaling. One of the key mediators in this axis is SCFAs, particularly butyrate [56]. It is produced as a result of fermentation of dietary fiber by commensal bacteria, such as *Faecalibacterium prausnitzii* and *Roseburia* spp. SCFAs have anti-inflammatory effects, strengthen the epithelial barrier, and influence the function of glial cells in the brain. Butyric acid may even cross the blood–brain barrier and regulate gene expression by HDAC, which promotes neuroplasticity and stress resistance [3]. The second important group of microbiome metabolites involved in nerve signaling are tryptophan derivatives, such as IPA and IAld [57]. They act as ligands for AHR and PXR receptors, which are responsible for immune balance in the intestines, barrier tightness, neuroimmune communication, and blood–brain barrier permeability. Lower levels of IPA were found in both IBD patients and people with depression, suggesting a common, microbiota-based disease fingerprint. The microbiota also influences tryptophan metabolism and serotonin production, as well as the kynurenine pathway, which is overactivated in chronic inflammation [58]. High levels of neurotoxic kynurenine metabolites have been observed in both IBD and mood disorders, which may explain symptoms such as depression, anxiety, or cognitive disorders. Importantly, similar microbiotic mechanisms are observed not only in IBD, but also in other diseases with inflammatory and neurological components, such as multiple sclerosis, Parkinson’s disease, autism spectrum disorders, and generalized anxiety disorders [59]. This shows that the microbiota does not function locally, but rather as a systemic regulator of neuroimmune health [60]. This concept paves the way for joint, targeted therapies for various diseases. In IBD, the link between the microbiome and mental disorders has particular clinical significance. Depression and anxiety are associated with greater disease activity, more frequent hospitalizations, poorer adherence to treatment recommendations, and a weaker response to biologic medications [61]. Treatment response remains heterogeneous, and emerging pharmaco-microbiomics research suggests that baseline microbiome features may contribute to variability in biologic effectiveness, although this requires further clinical validation. Chronic stress and co-occurring mental disorders exacerbate the inflammatory response by activating the hypothalamic–pituitary–adrenal (HPA) axis and sympathetic nervous system. New microbiome interventions may offer future therapeutic possibilities. Dietary strategies, especially the Mediterranean diet and a high-fiber diet, increase SCFAs production and improve mood in patients with chronic diseases [62]. Early research on so-called psychobiotics (probiotics that affect the central nervous system, such as *Lactiplantibacillus rhamnosus*, *Bifidobacterium longum*, and *Lactiplantibacillus helveticus*) indicates their potential in improving mood, regulating emotions, and cognitive function [63]. However, IBD-specific human data are limited; most clinical evidence comes from IBS or non-IBD cohorts, and results should be interpreted as preliminary.

In IBD, data are still limited, but the first clinical trials are emerging. Understanding the relationship between microbiota-derived metabolites, inflammation, and neuropsychiatric symptoms may inform future personalized adjunctive strategies addressing mental health in IBD. Biomarkers such as SCFAs profile, IPA concentration, or AHR pathway activity can be used as tools for risk stratification and targeted therapy. However, the most important thing is that a strategy restoring the diversity and functions of the microbiota can simultaneously improve the state of the intestines and mental health. Table 3 summarizes current evidence on psychobiotics relevant to IBD, highlighting their mechanisms of action within the gut–brain axis and their potential impact on both gastrointestinal and mental health outcomes.

However, caution is necessary, as much of the current evidence relies on preclinical murine models or studies in patients with irritable bowel syndrome. Thus, while the gut–brain axis offers a novel, experimental therapeutic target, the translation from bench to bedside requires rigorous validation before psychobiotics can be recommended as standard adjunctive therapy in IBD.

### 3.4. Extraintestinal Manifestations of IBD: Microbiota-Guided Pathophysiology and Organ-Specific Crosstalk

Extraintestinal manifestations (EIMs) occur in up to 40% of patients with IBD, affecting organs such as the liver, joints, eyes, skin, kidneys, and bones [64]. While traditionally viewed as immunologic complications of chronic gut inflammation, EIMs are increasingly recognized as part of a systemic immunometabolic syndrome, in which intestinal dysbiosis and microbial translocation play a central role. Disrupted epithelial barriers, abnormal immune cell trafficking, molecular mimicry, and modified microbial metabolite signaling are examples of mechanistic connections [65].

#### 3.4.1. The Gut–Liver Axis: PSC–IBD as a Microbiota-Driven Phenotype

Primary sclerosing cholangitis (PSC), a progressive cholangiopathy characterized by bile duct inflammation and fibrosis, is strongly associated with IBD, especially UC. This co-occurrence defines a unique clinical and microbial phenotype termed PSC–IBD [66]. Metagenomic studies reveal a characteristic microbial signature in these patients, including increased abundance of *Veillonella* and *Enterococcus* and reduced levels of butyrate-producing taxa, such as *Faecalibacterium prausnitzii* [67]. The gut–liver axis is maintained through enterohepatic circulation of BAs and microbial products. In PSC–IBD, impaired conversion of primary to secondary BAs due to reduced 7α-dehydroxylating bacteria, alters BAs profiles and attenuates FXR and TGR5 signaling in cholangiocytes and hepatic immune cells. This contributes to chronic inflammation, fibrosis, and a heightened risk of cholangiocarcinoma [68]. Regardless of IBD activity, recommendations for yearly colonoscopic surveillance from the time of PSC diagnosis are made due to the noteworthy increase in colorectal cancer (CRC) risk [69]. Hepatology co-management and early transplant referral are necessary in advanced cases because liver disease progression frequently happens independently of colonic activity [70].

#### 3.4.2. The Gut–Joint Axis: Microbial Dysbiosis and Spondyloarthropathy

Articular involvement is among the most prevalent EIMs in IBD, manifesting as both axial (e.g., sacroiliitis, ankylosing spondylitis) as well as peripheral arthritis. Peripheral arthritis is subclassified into Type 1 (pauciarticular, large joints, often flares with IBD activity) and Type 2 (polyarticular, small joints, independent course) [71]. Microbiota alterations in IBD-associated spondyloarthropathy include expansion of Enterobacteriaceae and depletion of *Faecalibacterium* and *Akkermansia*, correlating with gut barrier dysfunction and increased systemic translocation of PAMPs [72]. These PAMPs activate Th17 pathways and innate immune responses in synovial tissues. In HLA-B27-positive individuals, animal models demonstrate that microbial composition directly influences the emergence of arthritic phenotypes, likely through modulation of IL-17–mediated inflammation [73]. Therapeutic decisions depend on the type of arthritis. Gut-driven forms may respond to IBD control, while independent articular manifestations often require targeted immunomodulation such as anti-TNF agents [74].

#### 3.4.3. The Gut–Kidney Axis: Microbiota and Oxalate Stone Risk

Nephrolithiasis, particularly calcium oxalate stones, is more frequent in CD patients, especially after ileal or ileocolonic resections. Contributing factors include BAs malabsorption and fat maldigestion, which increase intestinal oxalate absorption in enteric hyperoxaluria [75]. Oxalobacter formigenes is a key commensal capable of degrading dietary oxalate. The absence of this species, often observed in IBD, is linked with higher urinary oxalate levels and stone formation. Prevention focuses on hydration and dietary oxalate management; microbiota-targeted re-colonization remains investigational [76]. Supplementary interventions, such as BAs sequestrants or potassium citrate, may be considered in high-risk cases.

#### 3.4.4. The Gut–Eye Axis: Immune Crosstalk and Ocular Inflammation

Ocular manifestations of IBD include episcleritis, scleritis, and anterior uveitis. Uveitis and scleritis frequently happen separately, but episcleritis frequently correlates with intestinal disease activity. This is a situation requiring urgent ophthalmological evaluation due to the risk of vision loss [77]. Immune crosstalk between the gut and the eye might be driven by translocation of microbial antigens, leading to activation of Th17 along with microglial responses within the blood–retina barrier. It has been shown that bacterial products, such as LPS, SCFAs, and tryptophan-derived indoles, influence microglial homeostasis and neuroimmune pathways [78]. According to recent research, metabolites associated with dysbiosis may affect the retinal pigment epithelium through pathways that are dependent on TLR4 and AHR, causing oxidative stress and tight junction degradation [79]. In murine models of colitis, gut inflammation was linked with increased permeability of the blood–retina barrier and enhanced expression of inflammatory markers such as IL-6, TNF-α, and MCP-1 in ocular tissues. Furthermore, a unique microbial signature has been identified in non-infectious uveitis patients, corroborating the theory of dysregulation of the gut-eye immune axis [80]. As of today, there is a lack of direct clinical evidence, but modulation of the microbiota using targeted postbiotics, probiotics such as *Lactiplantibacillus rhamnosus GG*, or anti-inflammatory diets might be utilized as a supplement to traditional local and systemic treatments. Given the high risk of irreversible visual impairment, additional research on gut-eye interactions and biomarker-based interventions may open up new diagnostic and therapeutic possibilities in patients with IBD-associated eye inflammation.

#### 3.4.5. The Gut–Skin Axis: Neutrophilic Dermatoses and Barrier Disruption

Cutaneous EIMs are heterogeneous and include erythema nodosum (EN), pyoderma gangrenosum (PG), hidradenitis suppurativa (HS), and psoriasis. EN typically correlates with intestinal flares, whereas PG and HS follow an independent course and reflect systemic immune dysregulation [80]. Recent studies suggest that gut and skin dysbiosis interact bidirectionally, affecting epithelial barriers, endotoxemia, and neutrophil activation. In particular, reduced SCFAs levels, altered tryptophan metabolism, and increased abundance of *Fusobacterium* and *Escherichia coli* have been implicated in skin inflammation [81]. Treatment requires biologic immunomodulation such as anti-TNF, IL-23 blockade, in conjunction with lifestyle interventions such as weight optimization, reduction in ultra-processed foods, and barrier support through diet.

#### 3.4.6. The Gut–Bone Axis: SCFAs, Tryptophan, and Bone Remodeling

One often neglected consequence of IBD is bone demineralization, which includes osteopenia and osteoporosis. Among the causes are vitamin shortages, corticosteroid use, and chronic inflammation [82]. By modifying the Receptor Activator of Nuclear Factor-κB Ligand/Osteoprotegerin (RANKL/OPG) axis and systemic cytokine profiles, SCFAs, especially butyrate, can inhibit osteoclastogenesis and encourage bone formation. Similarly, indole derivatives, by means of AHR activation, have an impact on bone marrow immunity and osteoblast signaling [83]. Insufficient microbial synthesis of these compounds could impair skeletal integrity. Gut bacterial makeup also affects vitamin D metabolism and absorption, therefore connecting dysbiosis with bone loss even further. Dual-energy X-ray absorptiometry (DXA) screening, reduction in corticosteroid exposure, high-fiber diets, sufficient calcium, protein, and vitamin D consumption, and resistance exercise are among clinical recommendations. Though experimental, methods targeting microbiota remain hopeful [84]. Overall, these organ-specific axes support EIMs as manifestations of systemic dysbiosis [85]. Table 4 summarizes the key microbiota–immune links and clinical implications.

## 4. Therapeutic Modulation of the Microbiota in IBD: Mechanisms, Efficacy, and Safety Concerns

### 4.1. Microbiota-Targeted Therapeutic Strategies in IBD

Knowing the part the gut microbiota plays in controlling intestinal and systemic immunity reveals fresh therapeutic options for the management of IBD [86]. Whereas conventional treatment aims at lowering inflammation brought on by the host’s immune system by immunosuppression, cytokine blockage, or leukocyte migration, the new approach is moving toward restoring balance in the intestine, which might imply causal instead of symptomatic care [87]. Three primary categories of therapies aimed at microbes are usually distinguished: restorative approaches [88], modulatory approaches [89], and defensive strategies [90]. Each of them differs in the degree of intervention, mechanism of action, and stage of clinical development. Restorative therapies are intended to rebuild the microbiota and bring back its metabolic processes and lost diversity. FMT, which involves transferring purified fecal matter from a healthy donor to the patient’s gastrointestinal tract in order to restore intestinal homeostasis, is the most well-researched example. Although this method is already accepted in the treatment of recurrent *C. difficile* infections, it has also shown therapeutic potential in ulcerative colitis [87]. Clinical trials, such as FOCUS and FMT-UC, have shown that in certain patients with active disease, repeated administrations from multiple donors can result in endoscopic and clinical remission. However, the donor profile has a significant impact on efficacy; so-called super-donors are distinguished by high microbial diversity and the presence of important taxa like *Roseburia* spp. and *Eubacterium hallii* [91]. Despite these promising observations, FMT in IBD remains experimental due to lack of standardization, the risk of infection, and variability of patient responses. To overcome these limitations, LBPs have been developed. These are defined microbial consortia or spore-based therapies that mimic the beneficial effects of FMT in a more controlled and safer manner [92]. One prominent example is SER-109 (VOWST™), an oral preparation of purified *Firmicutes* spores that prevents recurrence of *C. difficile*, including in patients with IBD, by restoring bile acid metabolism and activating FXR and TGR5 signaling pathways [89]. Another is REBYOTA^®^, a rectally administered preparation also approved for recurrent CDI. Both are contemporary, standardized forms of restorative therapy, and research is still being performed to determine how they might affect IBD. The second major therapeutic category comprises modulatory approaches, which do not replace the microbiota but rather influence its composition and activity. Diet plays a central role here: nutritional strategies such as the low fermentable oligosaccharides, disaccharides, monosaccharides and polyols (low-FODMAPs) diet, the Mediterranean diet, or the IBD-AID diet have been shown to promote SCFAs-producing bacteria, reduce inflammation, and improve well-being. Foods rich in fiber act as fermentation substrates, boosting the synthesis of butyrate and propionate, which fortify epithelial integrity and promote immunological tolerance [93]. Probiotics, defined as live microorganisms with health-promoting effects, have shown mixed efficacy in IBD. The best studied preparation, VSL#3, has demonstrated benefit in inducing and maintaining remission in pouchitis and in mild UC [94]. However, the effects are strain-dependent and often insufficient in the context of severe dysbiosis or active inflammation. In this light, new-generation probiotics such as *Faecalibacterium prausnitzii* and *Akkermansia muciniphila* are being investigated, as they may offer more targeted benefits [95]. Though data in IBD is still being gathered, prebiotics such as fructooligosaccharides (FOS) or inulin specifically encourage the growth of good bacteria, and their combination with probiotics are an interesting avenue. Finally, defensive strategies focus not on bacteria themselves but on their functional products. Postbiotics, which are metabolites or structural elements of biologically active microorganisms, are attracting interest as potentially safer and more accurate substitutes. These consist of secondary bile acids, indole derivatives, and SCFAs, all of which have the ability to improve Treg differentiation, lower inflammation, and fortify the epithelial barrier [96]. The delivery strategies being studied include encapsulated formulations intended to reach the inflamed colon or bacteria that have been engineered to produce particular metabolites. Particularly in patients with high inflammatory activity or immunosuppression, postbiotics may represent a promising option when the administration of live organisms is less desirable [97]. Personalized strategies based on microbiota profiling, metabolic biomarkers, and integration with established IBD therapies are likely to shape the future development of these defensive approaches. Table 5 categorizes and compares current microbiota-targeted therapeutic strategies in IBD, summarizing their mechanisms of action, clinical indications, and development status across restorative, modulatory, and defensive approaches.

### 4.2. Challenges, Risks and Future Directions in Microbiota-Based Therapies for IBD

Although interventions targeting the microbiota have great potential in the treatment of IBD, their use in everyday clinical practice still faces many obstacles. Like any new therapeutic class, it requires solid scientific evidence, appropriate diagnostic markers, and clear safety guidelines and regulations [98]. Although research on the microbiome is developing dynamically, establishing a direct causal link between dysbiosis and the development of IBD is still difficult. Most of the available data are correlational, we see changes in the microbiome composition, but it is not always clear whether they are the cause or effect of inflammation. Some bacteria, such as *Faecalibacterium prausnitzii,* are considered good, but the role of many other microorganisms and their metabolites remains unclear [99]. Additionally, the microbiota differs significantly between patients; it is influenced by genes, diet, medications, environment, and the disease itself. This makes the results of the research difficult to replicate in other groups, and the effectiveness of the therapy can vary greatly. Therefore, there is growing interest in researching the function of the microbiome, not just its composition, for example, what metabolites it produces and how they affect the host organism [100]. Although microbiome therapies are promising, they are not without risk. FMT, effective in treating recurrent *Clostridioides difficile* infection, may lead to complications in patients with IBD, such as disease exacerbation, bacteremia, or transmission of resistant bacterial strains [101]. A more modern approach, such as LBPs like SER-109 or REBYOTA^®^, appears to be safer and more predictable, but they are still being studied in the context of IBD and do not yet have formal indications for this use [102]. Moreover, many studies do not include long-term observation, so we do not know how durable the changes in the microbiota are and whether they may have later side effects, for example, a shift toward a microbiota that promotes inflammation or cancer. Another huge problem is the lack of predictive markers—we still do not know how to identify patients who will benefit the most from microbiotic therapy. Microbiome profiling, such as 16S rRNA sequencing, is promising, but it is not yet ready for routine clinical use [103]. There are no uniform rules governing microbiotic therapies yet. FMT is still considered an experimental procedure, except in emergency situations such as recurrent *C. difficile* infections. Although live biotherapeutics have been approved by the FDA for the treatment of rCDI, they require new studies and separate registration in IBD [104]. Microbiome therapies are an exciting new chapter in the treatment of IBD. However, their success will depend on solid translational research, good patient selection, clear regulations, and integration with current treatment methods. Over time, instead of being just an addition to therapy, they may become the foundation of personalized IBD treatment, targeting not only the symptoms, but the causes of the disease.

## 5. Conclusions

IBD exemplifies a systemic immunometabolic disorder in which dysbiosis and the loss of key microbial functions extend beyond the gut, contributing to recurrent infections, iron deficiency, neuropsychiatric comorbidities, and extraintestinal manifestations [4]. The incorporation of microbial metabolites, such as bile acids, tryptophan-derived indoles, and SCFAs, into the BMI loop concept emphasizes their crucial function in preserving host microbiota homeostasis and emphasizes how chronic inflammation is caused by disruption of this axis [23]. Innovative, causative approaches that aim to restore intestinal homeostasis beyond symptomatic management include probiotics, prebiotics, postbiotics, dietary modification, LBPs, fecal microbiota transplantation, and other microbiota-targeted methods [92]. Early clinical trials, especially in infection management and metabolic or neuroimmune regulation, demonstrate promising results, yet challenges remain in terms of patient heterogeneity, standardization, regulatory approval, and long-term safety [91]. Future research should focus on functional microbiome profiling, identification of predictive biomarkers, and precision-based interventions that integrate restorative, modulatory, and defensive approaches. Through this, therapies fueled by the microbiota may grow from experimental instruments to validated components of individualized IBD management, thereby potentially lowering disease activity, preventing complications, and improving patient outcomes overall.

## Figures and Tables

**Table 1:** Microbiota derived metabolites, their producers, host targets, and impact in IBD.

Metabolite Class	Key Producers	Main Targets	Biological Functions	Impact in IBD
SCFAs (butyrate, acetate, propionate)	*F. prausnitzii*, *Roseburia* spp., *Eubacterium* spp.	GPCRs (GPR41/43/109A), HDAC	- Fuel for colonocytes - Tight junctions - Treg induction - Anti-inflammatory cytokines	↓ SCFAs → impaired barrier, ↑ permeability, ↓ tolerance
Secondary BAs (DCA, LCA, isoDCA, 3-oxoLCA)	*C. scindens, C. hiranonis*	FXR, TGR5	- AMP induction - Th17/Treg balance - Epithelial regeneration	↓ 7α-dehydroxylation → ↓ immune modulation, ↑ inflammation
Tryptophan indoles (IPA, ILA, IAld)	*Lactiplantibacillus* spp., *Clostridium* spp., *Peptostreptococcus* spp.	AHR, PXR	- IL-22 signaling - Detox enzymes (CYP3A4, MDR1) - Mucosal repair - Treg activation	↓ Indoles → barrier dysfunction, loss of AHR, ↑ inflammation
Combined BMI loop (SCFAs + BAs + Indoles)	Multiple taxa	AHR, PXR, FXR, GPCRs	- Synergistic barrier/immune regulation - Colonization resistance - Homeostasis	Disruption → systemic inflammation, PAMPs translocation, chronic IBD

Abbreviations: SCFAs—short-chain fatty acids; DCA—deoxycholic acid; LCA—lithocholic acid; IPA—indole-3-propionic acid; ILA—indole-lactic acid; IAld—indole-3-aldehyde; AHR—aryl hydrocarbon receptor; PXR—pregnane X receptor; FXR—farnesoid X receptor; GPCR—G-protein-coupled receptor; Treg—regulatory T cell; HDAC—histone deacetylase; PAMPs—pathogen-associated molecular patterns. Symbols: ↓ indicates a reduction or depletion of the given metabolite or pathway activity; ↑ indicates an increase or enhancement.

**Table 2:** Mechanisms of dysbiosis in IBD, clinical consequences, and microbiota-based strategies.

Mechanism of Dysbiosis in IBD	Clinical Consequences	Microbiota-Based Strategies	Clinical Considerations
↓ SCFAs production (e.g., butyrate)	Impaired epithelial barrier, ↑ intestinal permeability, ↑ CRC risk	High-fiber diet, prebiotics, FMT	Also beneficial for mucosal immunity, Treg induction
↓ 7α-dehydroxylating bacteria (e.g., *Clostridium scindens*)	↓ Secondary bile acids → *C. difficile* germination and growth	SER-109, REBYOTA^®^, FMT	SER-109 contains purified *Firmicutes* spores
↑ PAMPs (e.g., LPS, flagellin, PGN)	Inflammatory amplification, impaired innate immunity	Restoration of eubiosis via FMT or probiotics	Associated with flare risk, chronic inflammation; NOD2 pathway relevant
↑ Intestinal permeability (‘leaky gut’)	Bacterial/toxin translocation → ↑ systemic inflammation	Postbiotics (e.g., SCFA, indoles), FMT	SCFAs act via GPCRs; indoles via AHR/PXR; enhances CDI susceptibility
Antibiotic or PPI exposure	Loss of colonization resistance, microbiome destabilization, reduced biologics efficacy	Minimized use; microbiota-supportive probiotics	Key risk factor for initial and recurrent CDI.
Recurrent CDI episodes	Difficult to treat, ↑ relapse, hospitalization risk	FMT, SER-109, REBYOTA^®^	FMT success rate >80% with proper patient selection; SER-109 and REBYOTA^®^ FDA-approved for rCDI

Abbreviations: SCFAs—Short-chain fatty acids; BAs—Bile acids; CRC—Colorectal cancer; PAMPs—Pathogen-associated molecular patterns; PGN—Peptidoglycan; NOD2—Nucleotide-binding oligomerization domain-containing protein 2; GPCR—G-protein-coupled receptor; AHR—Aryl hydrocarbon receptor; PXR—Pregnane X receptor; FXR—Farnesoid X receptor; TGR5—G-protein-coupled bile acid receptor 1; FMT—Fecal microbiota transplantation; CDI—*Clostridioides difficile* infection; LBP—Live biotherapeutic product; FDA—U.S. Food and Drug Administration. Symbols: ↓ indicates decreased production/abundance or reduced pathway activity; ↑ indicates increased levels/abundance or enhanced activity. Trademark notice: ^®^ denotes a registered trademark.

**Table 3:** Psychobiotics in IBD and gut–brain axis.

Psychobiotic Strain	Proposed Mechanism of Action	Observed Effects	Potential Role in IBD (Preliminary)	Reference
*Lactiplantibacillus helveticus R0052*	Modulation of GABA receptors; ↓ cortisol; immune regulation via cytokines	↓ anxiety and depression symptoms (Human RCT)	May alleviate stress-related flare-ups and improve resilience	[63]
*Bifidobacterium longum R0175*	SCFAs production; serotonin pathway modulation; ↓ systemic inflammation	Improved mood and reduced stress (clinical and preclinical studies)	Supports gut–brain balance; may improve quality of life in IBD	[63]
*Bifidobacterium longum 1714*	Modulation of prefrontal cortex activity; ↓ stress-induced cortisol	↓ perceived stress, improved cognitive performance (Human trial)	Neuroimmune regulation; may enhance treatment adherence	[62]
*Lactiplantibacillus rhamnosus JB-1*	GABAergic pathway activation via vagus nerve; modulation of HPA axis	Reduced anxiety-like behavior (Murine model)	May reduce psychological burden associated with chronic disease	[63]
*Lactiplantibacillus plantarum 299v*	Enhanced cognitive performance and decreased content of neurotoxic catabolites of tryptophan	↓ GI symptoms; ↑ iron bioavailability (Human RCT)	+ psychobiotic effects	[53]
*Bifidobacterium breve CCFM1025*	Kynurenine pathway modulation; anti-inflammatory cytokine balance	Antidepressant-like effects (Murine models; limited human data)	Emerging target for mood symptoms in inflammatory states	[53]

Abbreviations: SCFAs—Short-chain fatty acids; GABA—Gamma-aminobutyric acid; HPA—Hypothalamic–pituitary–adrenal; GI—Gastrointestinal; IBD—Inflammatory bowel disease; RCT—randomized controlled trial. Symbols: ↓ indicates a decrease or reduction; ↑ indicates an increase or enhancement.

**Table 4:** Organ axes in IBD: microbiota–immune–clinical links.

Organ Axis	Mechanistic Links	Microbiota Components	Clinical Implications
Gut–Liver (PSC–IBD)	Impaired bile acid metabolism; FXR/TGR5 dysregulation; microbial translocation	↑ *Veillonella*, ↓ F. *prausnitzii*, reduced secondary BAs	↑ Risk of cholangiocarcinoma & CRC. Action: annual colonoscopy (ECCO/AGA guidelines). Therapy FXR agonists under investigation
Gut–Joint	PAMPs translocation; Th17 activation; HLA-B27–microbiota interaction	↑ *Enterobacteriaceae*, ↓ *Faecalibacterium*, ↓ *Akkermansia*	Axial arthritis: less gut-dependent/Peripheral arthritis: ofter correlates with IBD activity. Therapy: JAK inhibitors or anti-TNF (tailored to subtype)
Gut–Kidney	Enteric hyperoxaluria; loss of oxalate-degrading bacteria	↓ *Oxalobacter formigenes*	↑ Risk of calcium oxalate stones. Action: hydration, calcium co-supplementation with meals. Experimental: oxalate-degrading probiotics
Gut–Eye	Disrupted gut barrier; Th17–microglia activation; neuroimmune signaling	LPS, SCFAs, tryptophan-derived indoles	Episcleritis: gut-related/Uveitis: independent course (urgent referral). Therapy: biologics, ophthalmologic monitoring.
Gut–Skin	Neutrophil activation; IL-1β/IL-8 signaling; systemic endotoxemia	↑ *Fusobacterium*, *E. coli*, ↓ SCFAs, altered indoles	Conditions: EN, PG, HS, psoriasis. Therapy: anti-TNF/JAKi/IL-23 blockade. Lifestyle: reduce ultra-processed foods, support barrier via fiber intake.
Gut–Bone	SCFAs/AHR-mediated immune modulation; vitamin D signaling dysregulation; corticosteroid effects	↓ Butyrate-producing bacteria, altered AHR ligands	Conditions: osteopenia, osteoporosis. Action: DXA screening, resistance training. Diet: Vitamin D + Calcium, high-fiber intake

Abbreviations: PSC—Primary sclerosing cholangitis; IBD—Inflammatory bowel disease; BAs—Bile acids; FXR—Farnesoid X receptor; TGR5—G-protein-coupled bile acid receptor 1; CRC—Colorectal cancer; PAMPs—Pathogen-associated molecular patterns; Th17—T helper 17 cells; HLA-B27—Human leukocyte antigen B27; TNF—Tumor necrosis factor; JAKi—Janus kinase inhibitors; SCFAs—Short-chain fatty acids; LPS—Lipopolysaccharide; EN—Erythema nodosum; PG—Pyoderma gangrenosum; HS—Hidradenitis suppurativa; UPF—Ultra-processed foods; AHR—Aryl hydrocarbon receptor; DXA—Dual-energy X-ray absorptiometry. Symbols: ↑ indicates increased abundance or activity; ↓ indicates decreased abundance or activity.

**Table 5:** Current and emerging microbiota-targeted therapeutic strategies in IBD.

Strategy	Mechanism of Action	Clinical Status	Potential Benefits in IBD
Fecal Microbiota Transplantation (FMT)	Restores microbial diversity and SCFAs production; suppresses inflammation via Treg induction	Experimental in IBD (standard of care for rCDI)	Clinical remission in UC; donor-dependent; safety under evaluation
Live Biotherapeutic Products (LBPs)	Defined microbial consortia restore colonization resistance and bile acid metabolism	FDA-approved for rCDI prevention (e.g., SER-109, REBYOTA^®^); investigational for IBD	May reduce infection risk, support mucosal healing
Diet (e.g., Mediterranean, CDED)	Enriches beneficial taxa; increases SCFAs; reduces dysbiosis and immune activation	Adjunctive therapy (supported by RCTs in mild-to-moderate IBD)	Improves symptoms and inflammation; supports microbiome diversity
Probiotics (e.g., LP299 v)	Compete with pathogens; produce SCFAs; modulate immunity	Approved for pouchitis; mixed data in UC/IBD	Maintenance therapy in select phenotypes; limited efficacy in flares
Next-gen probiotics (e.g., A. muciniphila)	Anti-inflammatory metabolites; barrier support; IL-10 induction	Preclinical to early clinical phase	Targeted immunoregulation; future precision application
Prebiotics (e.g., FOS, GOS, inulin)	Fuel for SCFAs-producing bacteria; indirect immune modulation	Early clinical studies	May enhance probiotic effects and microbiome resilience
Postbiotics (e.g., butyrate, IPA)	Direct delivery of microbial metabolites with immune and barrier effects	Experimental; formulations under development	Safe, controllable modulation of gut–immune axis

Abbreviations: FMT—Fecal microbiota transplantation; LBP—Live biotherapeutic product; RCT—Randomized controlled trial; UC—Ulcerative colitis; IBD—Inflammatory bowel disease; SCFAs—Short-chain fatty acids; FOS—Fructooligosaccharides; GOS—galactoologosaccharides; IPA—Indole-3-propionic acid; AHR—Aryl hydrocarbon receptor; GPCR—G-protein-coupled receptor; HDAC—Histone deacetylase. Trademark notice: ^®^ denotes a registered trademark.

## Data Availability

No new data were created or analyzed in this study.
